# Patient experiences: a qualitative systematic review of chemotherapy adherence

**DOI:** 10.1186/s12885-024-12353-z

**Published:** 2024-05-30

**Authors:** Amineh Rashidi, Susma Thapa, Wasana Sandamali Kahawaththa Palliya Guruge, Shubhpreet Kaur

**Affiliations:** School of Nursing and Midwifery, Edith Cowan University, 270 Joondalup Drive, Joondalup, Perth, WA 6027 Australia

**Keywords:** Cancer, Chemotherapy treatment, Medication adherence, Qualitative research, Patients experiences

## Abstract

Adherence to chemotherapy treatment is recognized as a crucial health concern, especially in managing cancer patients. Chemotherapy presents challenges for patients, as it can lead to potential side effects that may adversely affect their mobility and overall function. Patients may sometimes neglect to communicate these side effects to health professionals, which can impact treatment management and leave their unresolved needs unaddressed. However, there is limited understanding of how patients’ experiences contribute to improving adherence to chemotherapy treatment and the provision of appropriate support. Therefore, gaining insights into patients’ experiences is crucial for enhancing the accompaniment and support provided during chemotherapy.

This review synthesizes qualitative literature on chemotherapy adherence within the context of patients’ experiences. Data were collected from Medline, Web of Science, CINAHL, PsychINFO, Embase, Scopus, and the Cochrane Library, systematically searched from 2006 to 2023. Keywords and MeSH terms were utilized to identify relevant research published in English. Thirteen articles were included in this review. Five key themes were synthesized from the findings, including positive outlook, receiving support, side effects, concerns about efficacy, and unmet information needs. The review underscores the importance for healthcare providers, particularly nurses, to focus on providing comprehensive information about chemotherapy treatment to patients. Adopting recommended strategies may assist patients in clinical practice settings in enhancing adherence to chemotherapy treatment and improving health outcomes for individuals living with cancer.

**Supplementary Information:**

The online version contains supplementary material available at 10.1186/s12885-024-12353-z.

## Introduction

Cancer can affect anyone and is recognized as a chronic disease characterized by abnormal cell multiplication in the body [1]. While cancer is prevalent worldwide, approximately 70% of cancer-related deaths occur in low- to middle-income nations [1]. Disparities in cancer outcomes are primarily attributed to variations in the accessibility of comprehensive diagnosis and treatment among countries [1, 2]. Cancer treatment comes in various forms; however, chemotherapy is the most widely used approach [3]. Patients undergoing chemotherapy experience both disease-related and treatment-related adverse effects, significantly impacting their quality of life [4]. Despite these challenges, many cancer patients adhere to treatment in the hope of survival [5]. However, some studies have shown that concerns about treatment efficacy may hinder treatment adherence [6]. Adherence is defined as “the extent to which a person’s behaviour aligns with the recommendations of healthcare providers“ [7]. Additionally, treatment adherence is influenced by the information provided by healthcare professionals following a cancer diagnosis [8]. Patient experiences suggest that the decision to adhere to treatment is often influenced by personal factors, with family support playing a crucial role [8]. Furthermore, providing adequate information about chemotherapy, including its benefits and consequences, can help individuals living with cancer gain a better understanding of the advantages associated with adhering to chemotherapy treatment [9].

Recognizing the importance of adhering to chemotherapy treatment and understanding the impact of individual experiences of chemotherapy adherence would aid in identifying determinants of adherence and non-adherence that are modifiable through effective interventions [10]. Recently, systematic reviews have focused on experiences and adherence in breast cancer [11], self-management of chemotherapy in cancer patients [12], and the influence of medication side effects on adherence [13]. However, these reviews were narrow in scope, and to date, no review has integrated the findings of qualitative studies designed to explore both positive and negative experiences regarding chemotherapy treatment adherence. This review aims to synthesize the qualitative literature on chemotherapy adherence within the context of patients’ experiences.

## Methods

This review was conducted in accordance with the Joanna Briggs Institute [14] guidelines for systemic review involving meta-aggregation. This review was registered in PROSPERO (CRD42021270459).

### Search methods

The searches for peer reviewed publications in English from January 2006-September 2023 were conducted by using keywords, medical subject headings (MeSH) terms and Boolean operators ‘AND’ and ‘OR’, which are presented in the table in Appendix 1. The searches were performed in a systematic manner in core databases such including Embase, Medline, PsycINFO, CINAHL, Web of Science, Cochrane Library, Scopus and the Joanna Briggs Institute (JBI). The search strategy was developed from keywords and medical subject headings (MeSH) terms. Librarian’s support and advice were sought in forming of the search strategies.

### Study selection and inclusion criteria

The systematic search was conducted on each database and all articles were exported to Endnote and duplicates records were removed. Then, title and abstract of the full text was screened by two independent reviewers against the inclusion criteria. For this review, populations were patients aged 18 and over with cancer, the phenomenon of interest was experiences on chemotherapy adherence and context was considered as hospitals, communities, rehabilitation centres, outpatient clinics, and residential aged care. All peer-reviewed qualitative study design were also considered for inclusion. Studies included in this review were classified as primary research, published in English since 2006, some intervention implemented to improve adherence to treatment. This review excluded any studies that related to with cancer and mental health condition, animal studies and grey literature.

### Quality appraisal and data extraction

The JBI Qualitative Assessment and Review Instrument for qualitative studies was used to assess the methodological quality of the included studies, which was conducted by the primary and second reviewers independently. There was no disagreement between the reviews. The qualitative data on objectives, study population, context, study methods, and the phenomena of interest and findings form the included studies were extracted.

### Data synthesis

The meta-aggregation approach was used to combine the results with similar meaning. The primary and secondary reviewers created categories based on the meanings and concept. These categories were supported by direct quotations from participants. The findings were assess based on three levels of evidence, including unequivocal, credible, and unsupported [15, 16]. Findings with no quotation were not considered for synthesis in this review. The categories and findings were also discussed by the third and fourth reviewers until a consensus was reached. The review was approved by the Edith Cowan University Human Research Ethics Committee (2021–02896).

## Results

### Study inclusion

A total of 4145 records were identified through a systematic search. Duplicates (*n* = 647) were excluded. Two independent reviewers conducted screening process. The remaining articles (*n* = 3498) were examined for title and abstract screening. Then, the full text screening conducted, yielded 13 articles to be included in the final synthesis see Appendix 2.

### Methodological quality of included studies

All included qualitative studies scored between 7 and 9, which is displayed in Appendix 3. The congruity between the research methodology and the research question or objectives, followed by applying appropriate data collection and data analysis were observed in all included studies. Only one study [17] indicated the researcher’s statement regarding cultural or theoretical perspectives. Three studies [18–20] identified the influence of the researcher on the research and vice-versa.

### Characteristics of included studies

Most of studies conducted semi-structured and in-depth interviews, one study used narrative stories [19], one study used focus group discussion [21], and one study combined focus group and interview [22] to collect data. All studies conducted outpatient’s clinic, community, or hospital settings [17–29]. The study characteristics presented in Appendix 4.

### Review findings

Eighteen findings were extracted and synthesised into five categories: positive outlook, support, side effects, concern about efficacy and unmet information needs.

#### Positive outlook

Five studies discussed the link between positivity and hope and chemotherapy adherence [19, 20, 23, 27, 28]. Five studies commented that feeling positive and avoid the negativity and worry could encourage people to adhere in their mindset chemotherapy: “*I think the main thing for me was just keeping a positive attitude and not worrying, not letting myself worry about it*” [20]. Participants also considered the positive thoughts as a coping mechanism, that would help them to adhere and complete chemotherapy: “*I’m just real positive on how everything is going. I’m confident in the chemo, and I’m hoping to get out of her soon*” [23]. Viewing chemotherapy as part of their treatment regimen and having awareness of negative consequences of non-adherence to chemotherapy encouraged them to adhere chemotherapy: “*If I do not take medicine, I do not think I will be able to live*” [28]. Adhering chemotherapy was described as a survivor tool which helped people to control cancer-related symptoms: “*it is what is going to restore me. If it wasn’t this treatment, maybe I wasn’t here talking to you. So, I have to focus in what he is going to give me, life*!” [27]. Similarly, people accepted the medical facts and prevent their life from worsening; “*without the treatment, it goes the wrong way. It is hard, but I have accepted it from the beginning, yes. This is how it is. I cannot do anything about it. Just have to accept it*” [19].

#### Support

Finding from six studies contributed to this category [20, 21, 23–25, 29]. Providing support from families and friends most important to the people. Receiving support from family members enhanced a sense responsibility towards their families, as they believed to survive for their family even if suffered: “*yes, I just thought that if something comes back again and I say no, then I have to look my family and friends in the eye and say I could have prevented it, perhaps. Now, if something comes back again, I can say I did everything I could. Cancer is bad enough without someone saying: It’s your own fault!!”* [29]. Also, emotional support from family was described as important in helping and meeting their needs, and through facilitation helped people to adhere chemotherapy: “*people who genuinely mean the support that they’re giving […] just the pure joy on my daughter’s face for helping me. she was there day and night for me if I needed it, and that I think is the main thing not to have someone begrudgingly looking after you*” [20]. Another study discussed the role family, friends and social media as the best source of support during their treatment to adhere and continue “*I have tons of friends on Facebook, believe it or not, and it’s amazing how many people are supportive in that way, you know, just sending get-well wishes. I can’t imagine going through this like 10 years ago whenever stuff like that wasn’t around*” [23]. Receiving support from social workers was particularly helpful during chemotherapy in encouraging adherence to the chemotherapy: “*the social worker told me that love is courage. That was a huge encouragement, and I began to encourage myself*” [25].

#### Side effects

Findings from five studies informed this category [17, 21, 22, 25, 26]. Physical side effects were described by some as the most unpleasure experience: “*the side effects were very uncomfortable. I felt pain, fatigue, nausea, and dizziness that limited my daily activities. Sometimes, I was thinking about not keeping to my chemotherapy schedule due to those side effect*” [17]. The impact of side effects affected peoples’ ability to maintain their independence and self-care: “*I couldn’t walk because I didn’t have the energy, but I wouldn’t have dared to go out because the diarrhoea was so bad. Sometimes I couldn’t even get to the toilet; that’s very embarrassing because you feel like you’re a baby*” [26]. Some perceived that this resulted in being unable to perform independently: “*I was incredibly weak and then you still have to do things and you can’t manage it*” [22]. These side effect also decreased their quality of life “*I felt nauseated whenever I smelled food. I simply had no appetite when food was placed in front of me. I lost my sense of taste. Food had no taste anymore*” [25]. Although, the side effects impacted on patients´ leisure and free-time activities, they continued to undertake treatment: “*I had to give up doing the things I liked the most, such as going for walks or going to the beach. Routines, daily life in general were affected*” [21].

#### Concern about efficacy

Findings form four studies informed this category [17, 18, 24, 28]. Although being concerned about the efficacy of the chemotherapy and whether or not chemotherapy treatment would be successful, one participant who undertook treatment described: *“the efficacy is not so great. It is said to expect about 10% improvement, but I assume that it declines over time*” [28]. People were worried that such treatment could not cure their cancer and that their body suffered more due to the disease: “*I was really worried about my treatment effectiveness, and I will die shortly*” [17]. There were doubts expressed about remaining the cancer in the body after chemotherapy: “*there’s always sort of hidden worries in there that whilst they’re not actually taking the tumour away, then you’re wondering whether it’s getting bigger or what’s happening to it, whether it’s spreading or whatever, you know*” [24]. Uncertainty around the outcome of such treatment, or whether recovering from cancer or not was described as: *“it makes you feel confused. You don’t know whether you are going to get better or else whether the illness is going to drag along further”* [18].

#### Unmet information needs

Five studies contributed to this category [17, 21–23, 26]. The need for adequate information to assimilate information and provide more clarity when discussing complex information were described. Providing information from clinicians was described as minimal: *“they explain everything to you and show you the statistics, then you’re supposed to take it all on-board. You could probably go a little bit slower with the different kinds of chemo and grappling with these statistics”* [26]. People also used the internet search to gain information about their cancer or treatments, *“I’ve done it (consult google), but I stopped right away because there’s so much information and you don’t know whether it’s true or not*” [21]. The need to receive from their clinicians to obtain clearer information was described as” *I look a lot of stuff up online because it is not explained to me by the team here at the hospital*” [23]. Feeling overwhelmed with the volume of information could inhibit people to gain a better understanding of chemotherapy treatment and its relevant information: “*you don’t absorb everything that’s being said and an awful lot of information is given to you*” [22]. People stated that the need to know more information about their cancer, as they were never dared to ask from their clinicians: “*I am a low educated person and come from a rural area; I just follow the doctor’s advice for my health, and I do not dare to ask anything”* [17].

## Discussion

The purpose of this review was to explore patient’s experiences about the chemotherapy adherence. After finalizing the searches, thirteen papers were included in this review that met the inclusion criteria.

The findings of the present review suggest that social support is a crucial element in people’s positive experiences of adhering to chemotherapy. Such support can lead to positive outcomes by providing consistent and timely assistance from family members or healthcare professionals, who play vital roles in maintaining chemotherapy adherence [30]. Consistent with our study, previous research has highlighted the significant role of family members in offering emotional and physical support, which helps individuals cope better with chemotherapy treatment [31, 32]. However, while receiving support from family members reinforces individuals’ sense of responsibility in managing their treatment and their family, it also instils a desire to survive cancer and undergo chemotherapy. One study found that assuming self-responsibility empowers patients undergoing chemotherapy, as they feel a sense of control over their therapy and are less dependent on family members or healthcare professionals [33]. A qualitative systematic review reported that support from family members enables patients to become more proactive and effective in adhering to their treatment plan [34]. This review highlights the importance of maintaining a positive outlook and rational beliefs as essential components of chemotherapy adherence. Positive thinking helps individuals recognize their role in chemotherapy treatment and cope more effectively with their illness by accepting it as part of their treatment regimen and viewing it as a tool for survival. This finding is supported by previous studies indicating that positivity and positive affirmations play critical roles in helping individuals adapt to their reality and construct attitudes conducive to chemotherapy adherence [35, 36]. Similarly, maintaining a positive mindset can foster more favourable thoughts regarding chemotherapy adherence, ultimately enhancing adherence and overall well-being [37].

This review identified side effects as a significant negative aspect of the chemotherapy experience, with individuals expressing concerns about how these side effects affected their ability to perform personal self-care tasks and maintain independent living in their daily lives. Previous studies have shown that participants with a history of chemotherapy drug side effects were less likely to adhere to their treatment regimen due to worsening symptoms, which increased the burden of medication side effects [38, 39]. For instance, cancer patients who experienced minimal side effects from chemotherapy were at least 3.5 times more likely to adhere to their treatment plan compared to those who experienced side effects [40]. Despite experiencing side effects, patients were generally willing to accept and adhere to their treatment program, although one study in this review indicated that side effects made some patients unable to maintain treatment adherence. Side effects also decreased quality of life and imposed restrictions on lifestyle, as seen in another study where adverse effects limited individuals in fulfilling daily commitments and returning to normal levels of functioning [41]. Additionally, unmet needs regarding information on patients’ needs and expectations were common. Healthcare professionals were considered the most important source of information, followed by consultation with the internet. Providing information from healthcare professionals, particularly nurses, can support patients effectively and reinforce treatment adherence [42, 43]. Chemotherapy patients often preferred to base their decisions on the recommendations of their care providers and required adequate information retention. Related studies have highlighted that unmet needs among cancer patients are known factors associated with chemotherapy adherence, emphasizing the importance of providing precise information and delivering it by healthcare professionals to improve adherence [44, 45]. Doubts about the efficacy of chemotherapy treatment, as the disease may remain latent, were considered negative experiences. Despite these doubts, patients continued their treatment, echoing findings from a study where doubts regarding efficacy were identified as a main concern for chemotherapy adherence. Further research is needed to understand how doubts about treatment efficacy can still encourage patients to adhere to chemotherapy treatment.

### Strengths and limitation

The strength of this review lies in its comprehensive search strategy across databases to select appropriate articles. Additionally, the use of JBI guidelines provided a comprehensive and rigorous methodological approach in conducting this review. However, the exclusion of non-English studies, quantitative studies, and studies involving adolescents and children may limit the generalizability of the findings. Furthermore, this review focuses solely on chemotherapy treatment and does not encompass other types of cancer treatment.

## Conclusion and practical implications

Based on the discussion of the findings, it is evident that maintaining a positive mentality and receiving social support can enhance chemotherapy adherence. Conversely, experiencing treatment side effects, concerns about efficacy, and unmet information needs may lead to lower adherence. These findings present an opportunity for healthcare professionals, particularly nurses, to develop standardized approaches aimed at facilitating chemotherapy treatment adherence, with a focus on providing comprehensive information. By assessing patients’ needs, healthcare professionals can tailor approaches to promote chemotherapy adherence and improve the survival rates of people living with cancer. Raising awareness and providing education about cancer and chemotherapy treatment can enhance patients’ understanding of the disease and its treatment options. Utilizing videos and reading materials in outpatient clinics and pharmacy settings can broaden the reach of educational efforts. Policy makers and healthcare providers can collaborate to develop sustainable patient education models to optimize patient outcomes in the context of cancer care. A deeper understanding of individual processes related to chemotherapy adherence is necessary to plan the implementation of interventions effectively. Further research examining the experiences of both adherent and non-adherent patients is essential to gain a comprehensive understanding of this topic.

### Electronic supplementary material

Below is the link to the electronic supplementary material.

Supplementary Material 1

Supplementary Material 2

Supplementary Material 3

Supplementary Material 4

Supplementary Material 5

## Data Availability

The datasets used and/or analysed during the current study available from the corresponding author on reasonable request. on our submission system as well.
