# Supplementary material for: Patient experiences: a qualitative systematic review of chemotherapy adherence
Source: BMC Cancer. 2024 May 30;24:658. doi: 10.1186/s12885-024-12353-z (PMC11138062; doi:10.1186/s12885-024-12353-z)
Supplement: Supplementary file 2 — Supplementary Material 2 [file 12885_2024_12353_MOESM2_ESM.pdf]

APPENDIX 2

Study selection and Prisma flow diagram

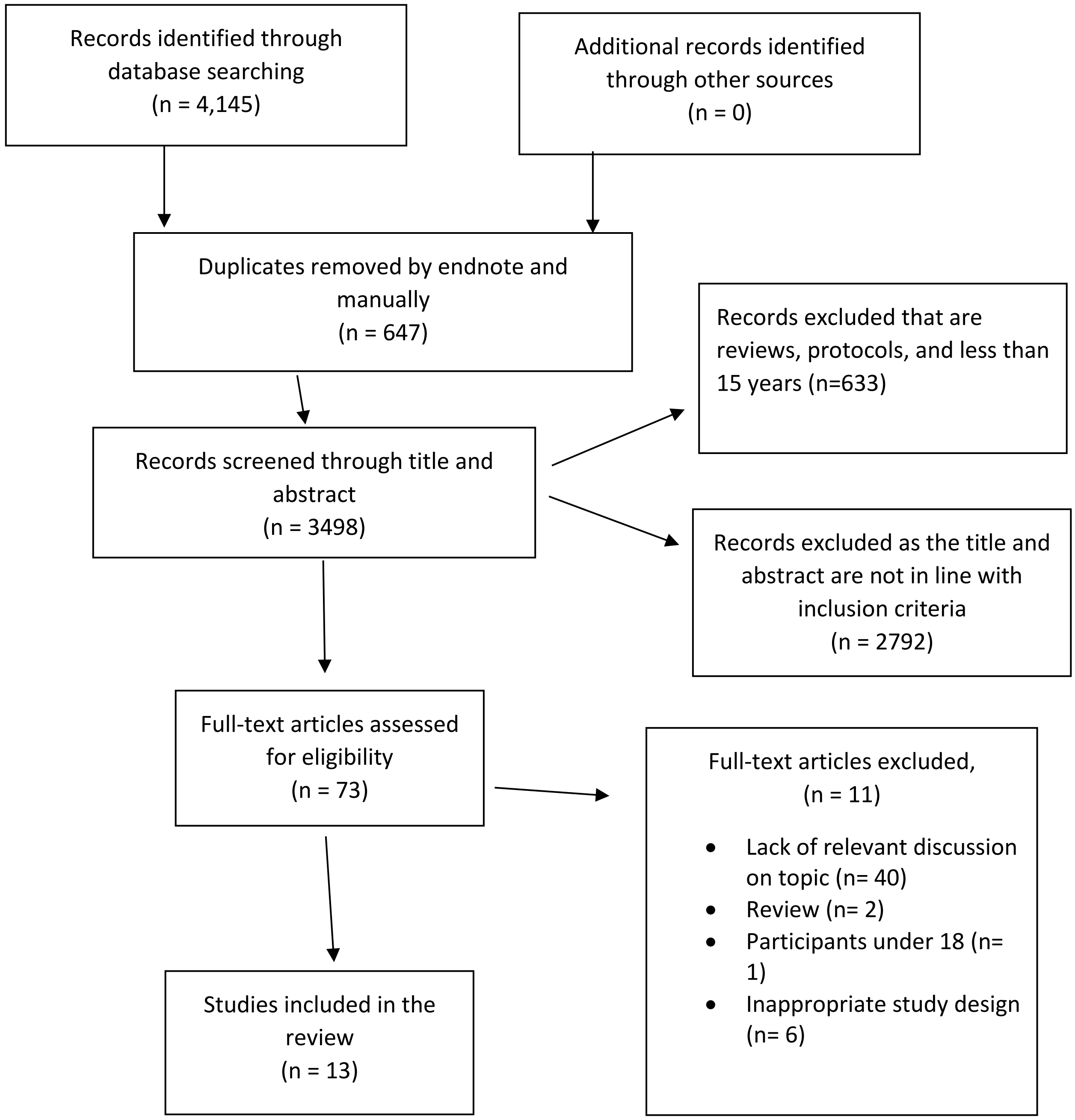

From: Moher D, Liberati A, Tetzlaff J, Altman DG, The PRISMA Group (2009). *P*referred *R*eporting *I*tems for *S*ystematic Reviews and *M*eta-*A*nalyses: The PRISMA Statement. PLoS Med 6(7): e1000097. doi:10.1371/journal.pmed1000097
