# Supplementary material for: Patient experiences: a qualitative systematic review of chemotherapy adherence
Source: BMC Cancer. 2024 May 30;24:658. doi: 10.1186/s12885-024-12353-z (PMC11138062; doi:10.1186/s12885-024-12353-z)
Supplement: Supplementary file 3 — Supplementary Material 3 [file 12885_2024_12353_MOESM3_ESM.pdf]

APPENDIX 1

Search Strategy

CINAHL (Filtered for 2006-2021 and English)

(("patients with cancer") OR ("cancer patient*") OR (cancer sufferers) OR (oncology patients) OR (cancer survivor*) OR ((MM "Cancer Patients"))) OR ("Neoplasms") AND (((MH "Chemotherapy, Cancer+")) OR chemo OR chemotherapy OR ("chemotherapy treatment") OR ("chemotherapy adherence") OR("medication adherence") OR ("anti-cancer adherence") OR ("anti-neoplastic") OR ("chemotherapy experience") OR ("chemotherapy perception") OR ((MM "Chemotherapy, Cancer"))) AND (hospital OR ("hospital setting"))

MEDLINE (Filtered for 2006-2021 and English)

(("patients with cancer") OR ("cancer patients") OR (cancer sufferers) OR (oncology patients) OR ("cancer patient experience")) OR ("Neoplasms") AND (chemotherapy OR ("chemotherapy adherence") OR ("medication adherence") OR (anti-cancer adherence) OR ("anti-neoplastic") OR ("chemotherapy experiences") OR (chemotherapy perception) OR ("chemotherapy treatment") OR ("patients undergoing chemotherapy")) AND (hospital OR communities)

EMBASE (Filtered for 2006-2021 and English)

(('cancer patients' OR (('cancer'/exp OR cancer) AND ('patients'/exp OR patients)) OR 'patients with cancer' OR (('patients'/exp OR patients) OR ("Neoplasms") AND with AND ('cancer'/exp OR cancer)) OR 'cancer survivor'/exp OR 'cancer survivor' OR (('cancer'/exp OR cancer) AND ('survivor'/exp OR survivor)) OR 'cancer sufferers'/exp OR 'cancer sufferers' OR (('cancer'/exp OR cancer) AND ('sufferers'/exp OR sufferers))) AND ('chemotherapy'/exp OR chemotherapy) OR chemo OR 'chemotherapy treatment' OR anti-neoplastic (('chemotherapy'/exp OR chemotherapy) AND ('treatment'/exp OR treatment)) OR 'chemotherapy adherence' OR ("medication adherence") OR (anti-cancer adherence) (('chemotherapy'/exp OR chemotherapy) AND ('adherence'/exp OR adherence))) AND ('patients experience' OR (('patients'/exp OR patients) AND ('experience'/exp OR experience))) OR 'chemotherapy perception' OR (('chemotherapy'/exp OR chemotherapy) AND ('perception'/exp OR perception)) OR 'chemotherapy experience' OR (('chemotherapy'/exp OR chemotherapy) AND ('experience'/exp OR experience)) #3 AND ([adult]/lim OR [aged]/lim

OR [very elderly]/lim) AND 'article'/it AND (2006:py OR 2007:py OR 2008:py OR 2009:py OR 2010:py OR 2011:py OR 2012:py OR 2013:py OR 2014:py OR 2015:py OR 2016:py OR 2017:py OR 2018:py OR 2019:py OR 2020:py OR 2021:py)

PsycINFO (Filtered for 2006-2021 and English)

(("cancer patients") OR ("patients with cancer") OR ("oncology patients") OR (Cancer sufferer) OR (Cancer survivors) OR ("chemotherapy patients")) OR ("Neoplasms") AND (Chemotherapy OR (Chemotherapy treatment) OR (medication adherence) OR (anti-cancer adherence) OR Chemo OR ("anti-neoplastic") ("Chemotherapy experience") OR ("Chemotherapy perception") OR ("Chemotherapy adherence") OR (MM "Chemotherapy")) AND ((cancer clinics) OR (cancer hospital) OR ("cancer in communities"))

Web of Science (Filtered through 2006-2021 and English)

("Cancer patients" OR cancer OR "patients with cancer" OR oncology patients OR Neoplasms AND chemotherapy OR chemotherapy adherence OR ("medication adherence") OR (anti-cancer adherence) OR "anti-neoplastic" "patients with chemotherapy" OR chemotherapy experiences OR "Chemotherapy patients experiences" AND hospital OR "cancer outpatients clinics" Communities)
