# Supplementary material for: Patient experiences: a qualitative systematic review of chemotherapy adherence
Source: BMC Cancer. 2024 May 30;24:658. doi: 10.1186/s12885-024-12353-z (PMC11138062; doi:10.1186/s12885-024-12353-z)
Supplement: Supplementary file 4 — Supplementary Material 4 [file 12885_2024_12353_MOESM4_ESM.pdf]

APPENDIX 3

Qualitative Quality Items

Citation	Q1	Q2	Q3	Q4	Q5	Q6	Q7	Q8	Q9	Q10	Score/10
(1)	Y	Y	Y	Y	Y	U	N	Y	Y	Y	8/10
(2)	Y	Y	Y	Y	Y	N	Y	Y	Y	Y	9/10
(3)	Y	Y	Y	Y	Y	N	U	Y	Y	Y	8/10
(4)	Y	Y	Y	Y	Y	N	N	Y	Y	Y	8/10
(5)	Y	Y	Y	Y	Y	N	N	Y	Y	Y	8/10
(6)	Y	Y	Y	Y	Y	U	U	Y	Y	Y	8/10
(7)	Y	Y	Y	Y	Y	U	Y	Y	Y	Y	8/10
(8)	Y	Y	Y	Y	Y	U	Y	Y	Y	Y	9/10
(9)	Y	Y	Y	Y	Y	U	N	Y	Y	U	7/10
(10)	Y	Y	Y	Y	Y	N	N	Y	Y	U	7/10
(11)	Y	Y	Y	Y	Y	N	U	Y	Y	Y	8/10
(12)	Y	Y	Y	Y	Y	N	U	Y	U	Y	7/10
(13)	Y	Y	Y	Y	Y	Y	U	Y	Y	Y	9/10

Y: Yes, N: No, U: Unclear

Questions:

1. Is there congruity between the stated philosophical perspective and the research methodology?
2. Is there congruity between the research methodology and the research question or objectives?
3. Is there congruity between the research methodology and the methods used to collect data?
4. Is there congruity between the research methodology and the representation and analysis of data?
5. Is there congruity between the research methodology and the interpretation of results?
6. Is there a statement locating the researcher culturally or theoretically?
7. Is the influence of the researcher on the research, and vice- versa, addressed?
8. Are participants, and their voices, adequately represented?
9. Is the research ethical according to current criteria or, for recent studies, and is there evidence of ethical approval by an appropriate body?
10. Do the conclusions drawn in the research report flow from the analysis, or interpretation, of the data?

1. Albrecht TA, Keim-Malpass J, Boyiadzis M, Rosenzweig M. Psychosocial experiences of young adults diagnosed with acute leukemia during hospitalization for induction chemotherapy treatment. Journal of Hospice & Palliative Nursing. 2019;21(2):167-73.
2. Chircop D, Scerri J. The lived experience of patients with non-Hodgkin's lymphoma undergoing chemotherapy. European Journal of Oncology Nursing. 2018;35:117-21.

3. Chou J-F, Lu YY. Intraperitoneal chemotherapy: The lived experiences of Taiwanese patients with ovarian cancer. Clinical Journal of Oncology Nursing. 2019;23(6):E100-E6.
4. Dumas L, Lidington E, Appadu L, Jupp P, Husson O, Banerjee S, et al. Exploring older women's attitudes to and experience of treatment for advanced ovarian cancer: A qualitative phenomenological study. Cancers. 2021;13(6):1207.
5. Farrell C, Heaven C. Understanding the impact of chemotherapy on dignity for older people and their partners. European Journal of Oncology Nursing. 2018;36:82-8.
6. Gassmann C, Kolbe N, Brenner A. Experiences and coping strategies of oncology patients undergoing oral chemotherapy: First steps of a grounded theory study. European Journal of Oncology Nursing. 2016;23:106-14.
7. Kvåle K, Synnes O. Living with life-prolonging chemotherapy—control and meaning-making in the tension between life and death. European Journal of Cancer Care. 2018;27(1):1-.
8. Staneva AA, Beesley VL, Niranjan N, Gibson AF, Rowlands I, Webb PM. "I wasn't gonna let it stop me": Exploring women's experiences of getting through chemotherapy for ovarian cancer. Cancer Nursing. 2019;42(2):E31-E8.
9. Talens A, Guilabert M, Lumbreras B, Aznar MT, López-Pintor E. Medication Experience and Adherence to Oral Chemotherapy: A Qualitative Study of Patients' and Health Professionals' Perspectives. International journal of environmental research and public health. 2021;18(8).
10. Wakiuchi J, Silva Marcon S, de Oliveira DC, Aparecida Sales C. Rebuilding subjectivity from the experience of cancer and its treatment. Revista Brasileira de Enfermagem. 2019;72(1):125-33.
11. Beaver K, Williamson S, Briggs J. Exploring patient experiences of neo-adjuvant chemotherapy for breast cancer. European Journal of Oncology Nursing. 2016;20:77-86.
12. Yagasaki K, Komatsu H, Takahashi T. Inner conflict in patients receiving oral anticancer agents: a qualitative study. BMJ Open [Internet]. 2015; 5(4).
13. Iskandarsyah A, de Klerk C, Suardi DR, Soemitro MP, Sadarjoen SS, Passchier J. Psychosocial and Cultural Reasons for Delay in Seeking Help and Nonadherence to Treatment in Indonesian Women With Breast Cancer: A Qualitative Study. Health Psychology. 2014;33(3):214-21.
