# Supplementary material for: Patient experiences: a qualitative systematic review of chemotherapy adherence
Source: BMC Cancer. 2024 May 30;24:658. doi: 10.1186/s12885-024-12353-z (PMC11138062; doi:10.1186/s12885-024-12353-z)
Supplement: Supplementary file 5 — Supplementary Material 5 [file 12885_2024_12353_MOESM5_ESM.pdf]

APPENDIX 4

Characteristics of included studies for methodological review

**Study**	**Methods for data collection and analysis**	**Country**	**Topic/ Phenomenon of interest**	**Setting/context /culture**	**Participants Characteristics and sample size**	**Main results**
(1)	Qualitative longitudinal study. Convenience sample.<br>Semi-structured interviews.<br>Qualitative descriptive analysis with inductive thematic development guide.	University of Pittsburgh, Pennsylvania, USA	Psychosocial Experiences of Young Adults Diagnosed with Acute Leukemia During Hospitalization for Induction Chemotherapy Treatment	Inpatient hematology oncology unit of a quaternary academic medical centre.	Young adults, aged between 18-39 years.<br>7 participants (4 females, 3 males).	Three themes emerged: getting through, supported yet isolated and information exchange preferences.
(2)	Qualitative research, Interpretative phenomenological analysis.<br>Purposive sampling.	London, UK	The lived experience of patients with non-Hodgkin's lymphoma	On haematology ward	Six participants (4 males and 2 females) age ranged from 44-81 years.	Three super-ordinate themes emerged: ‘Living an emotional rollercoaster’, ‘Becoming dependent on others’, and ‘Facing an uncertain future’.

	Semi-structured interviews and thematic analysis.		undergoing chemotherapy			
(3)	Descriptive qualitative study. Convenience sampling. Semi-structured interview. Content analysis inductive approach.	Taiwan	The lived experiences of Taiwanese patients with ovarian cancer	Hospital	9 participants aged above 20 years	Seven themes:<br>Feeling extreme anxiety and uneasiness,<br>Experiencing specific symptoms,<br>Experiencing unexpected or severe physical issues and psychological discomfort,<br>Feeling isolated from people,<br>Learning how to manage side effects, Worrying about families, illness progression, and the future,<br>Receiving support from others and accepting the illness.
(4)	Purposive sampling. Interview and focus group discussion. Thematic analysis.	London, UK	Exploring Older Women's Attitudes to and Experience of Treatment for Advanced Ovarian	Hospital	15 women. Women aged 65 years and above	Themes are:<br>1.Multifactorial decision-making.<br>1.1 Reception and retention of information cloud decision.<br>1.2 lengthening life expectancy.

			Cancer: A Qualitative Phenomenological Study			1.3 family influence<br>2. Burden of logistical issues<br>2.1 care coordination.<br>2.2 transport and informal support<br>3. Side-effects<br>3.1 weighed down by side-effects.<br>3.2 determination not to let cancer interfere
(5)	Qualitative descriptive method.<br>Semi-structured interviews.<br>Thematic analysis.	United Kingdom , England	Understanding the impact of chemotherapy on dignity for older people and their partners	Regional cancer center	20 patients (6 males, 14 females) and 10 partners (8 males, 2 females).<br>Aged 65-81 years.	Five main themes were associated with dignity: managing chemotherapy side-effects, personal feelings, maintaining independence, stoicism, and 'being lucky'.
(6)	Qualitative research, grounded theory.<br>Open interview. Data collection and analysis followed the principles	Switzerla nd	Experiences and coping strategies of oncology patients undergoing oral chemotherapy: First	Oncological outpatient clinic of an urban Swiss hospital.	Six patients and two spouses (5 males, 1 females)	The participants reported physical and emotional reluctance towards oral chemotherapy as well as toxic side effects. Feeling responsible emerged as a core phenomenon.

	of Straussian grounded theory.		steps of a grounded theory study			
(7)	Qualitative study. Narrative study. Purposive, non-probability sampling. Thematic narrative analysis.	Norway	Living with life-prolonging chemotherapy—control and meaning-making in the tension between life and death	Outpatient clinic of oncology department	13 participants (6 males, 7 females)	. The main findings showed that the narrators considered their lives worth living in spite of the treatment. They seemed to take control and build a new life on “what was left after the storm,” and described how they found meaning living in the tension between life and death.
(8)	a qualitative approach within a critical realist framework. Semi-structured interviews. Critical realist theoretical approach and thematic analysis.	Brisbane, Australia	I Wasn’t Gonna Let It Stop Me: Exploring Women’s Experiences of Getting Through Chemotherapy for Ovarian Cancer	Hospital	18 women. Aged 18 years and above.	3 main themes related to women"s experiences of dealing with chemotherapy: ’’optimistic tenacity,’’ which illustrates a specific stoic identity that women assumed during treatment; ’’self-care,’’ which reflects the health behaviors and activities women engaged in and lifestyle

						adjustments they made; and ’’support systems,’’ which emphasizes the importance of social, emotional, and medical support and the specific needs shared by women undergoing treatment for ovarian cancer
(9)	Qualitative study. Snowball sampling. Focus group discussion. Thematic analysis.	Spain	Medication Experience and Adherence to Oral Chemotherapy: A Qualitative Study of Patients’ and Health Professionals’ Perspectives	Hospital	23 Patients (7 males, 16 females) and 18 healthcare professionals. Aged ranged from 32-81 years.	The most impactful aspects in patients’ ME were the presence of adverse effects; lack of information about treatment; beliefs, needs and expectations regarding medications; social and family support; and the relationship with the health professionals.
(10)	Descriptive qualitative study. Semi-structured interviews. Thematic categorical content analysis.	Brazil	Rebuilding subjectivity from the experience of cancer and its treatment	Oncology care center, hospital	29 patients (23 females, 6 males)	Four categories have emerged denoting attitudes, feelings and experiences associated with chemotherapy and the need for reconstruction of daily life,

						permeated by the distancing of social life and work.
(11)	Qualitative approach.<br>In-depth face-to-face Interviews.<br>Thematic content analysis.	United Kingdom, England	Exploring patient experiences of neo-adjuvant chemotherapy for breast cancer	Four hospitals	20 participants	Five main themes emerged: coping with the rapid transition from ‘well’ to ‘ill’, information needs and decision making, needing support and empathy, impact on family, and creating a new ‘normal’.
(12)	Qualitative study, grounded theory approach.<br>Semi-structured interviews.<br>Data analyzed based on grounded theory methodology.	Japan	Inner conflict in patients receiving oral anticancer agents: a qualitative study	Hospital	14 patients.<br>Aged ranged from 43-80 years.	Patients with cancer experienced inner conflict between rational belief and emotional resistance to taking medication due to confrontation with cancer, doubt regarding efficacy and concerns over potential harm attached to use of the agent.
(13)	Qualitative study.<br>Purposive sampling.<br>Semi-structured interviews.	Indonesia	Psychosocial and Cultural Reasons for Delay in Seeking Help and Nonadherence to	Oncology outpatient clinic, hospital	50 participants.<br>Age ranged 34-65 years.	Eight main themes concerning reasons for delay in seeking medical help and treatment nonadherence emerged, namely: lack of awareness and knowledge,

	Data analysed based on grounded theory approach (thematic analysis).		Treatment in Indonesian Women with Breast Cancer: A Qualitative Study			cancer beliefs, treatment beliefs, financial problems, emotional burden, severe side effects, paternalistic style of communication, and unmet information needs

1. Albrecht TA, Keim-Malpass J, Boyiadzis M, Rosenzweig M. Psychosocial experiences of young adults diagnosed with acute leukemia during hospitalization for induction chemotherapy treatment. Journal of Hospice & Palliative Nursing. 2019;21(2):167-73.
2. Chircop D, Scerri J. The lived experience of patients with non-Hodgkin's lymphoma undergoing chemotherapy. European Journal of Oncology Nursing. 2018;35:117-21.
3. Chou J-F, Lu YY. Intraperitoneal chemotherapy: The lived experiences of Taiwanese patients with ovarian cancer. Clinical Journal of Oncology Nursing. 2019;23(6):E100-E6.
4. Dumas L, Lidington E, Appadu L, Jupp P, Husson O, Banerjee S, et al. Exploring older women's attitudes to and experience of treatment for advanced ovarian cancer: A qualitative phenomenological study. Cancers. 2021;13(6):1207.
5. Farrell C, Heaven C. Understanding the impact of chemotherapy on dignity for older people and their partners. European Journal of Oncology Nursing. 2018;36:82-8.
6. Gassmann C, Kolbe N, Brenner A. Experiences and coping strategies of oncology patients undergoing oral chemotherapy: First steps of a grounded theory study. European Journal of Oncology Nursing. 2016;23:106-14.
7. Kvåle K, Synnes O. Living with life-prolonging chemotherapy—control and meaning-making in the tension between life and death. European Journal of Cancer Care. 2018;27(1):1-.
8. Staneva AA, Beesley VL, Niranjan N, Gibson AF, Rowlands I, Webb PM. "I wasn't gonna let it stop me": Exploring women's experiences of getting through chemotherapy for ovarian cancer. Cancer Nursing. 2019;42(2):E31-E8.
9. Talens A, Guilabert M, Lumbreras B, Aznar MT, López-Pintor E. Medication Experience and Adherence to Oral Chemotherapy: A Qualitative Study of Patients' and Health Professionals' Perspectives. International journal of environmental research and public health. 2021;18(8).

10. Wakiuchi J, Silva Marcon S, de Oliveira DC, Aparecida Sales C. Rebuilding subjectivity from the experience of cancer and its treatment. Revista Brasileira de Enfermagem. 2019;72(1):125-33.
11. Beaver K, Williamson S, Briggs J. Exploring patient experiences of neo-adjuvant chemotherapy for breast cancer. European Journal of Oncology Nursing. 2016;20:77-86.
12. Yagasaki K, Komatsu H, Takahashi T. Inner conflict in patients receiving oral anticancer agents: a qualitative study. BMJ Open [Internet]. 2015; 5(4).
13. Iskandarsyah A, de Klerk C, Suardi DR, Soemitro MP, Sadarjoen SS, Passchier J. Psychosocial and Cultural Reasons for Delay in Seeking Help and Nonadherence to Treatment in Indonesian Women With Breast Cancer: A Qualitative Study. Health Psychology. 2014;33(3):214-21.
